# Orphan nuclear receptor TLX regulates astrogenesis by modulating BMP signaling

**DOI:** 10.3389/fnins.2014.00074

**Published:** 2014-04-10

**Authors:** Song Qin, Wenze Niu, Nida Iqbal, Derek K. Smith, Chun-Li Zhang

**Affiliations:** ^1^Center for Translational Neurodegeneration and Regenerative Therapy, Shanghai Tenth People's Hospital, Tongji University School of MedicineShanghai, China; ^2^Department of Molecular Biology, University of Texas Southwestern Medical CenterDallas, TX, USA

**Keywords:** nuclear receptor, TLX, neural stem cells, neurogenesis, astrogenesis, BMP-SMAD signaling

## Abstract

Neural stem cells (NSCs) are self-renewing multipotent progenitors that generate both neurons and glia. The precise control of NSC behavior is fundamental to the architecture and function of the central nervous system. We previously demonstrated that the orphan nuclear receptor TLX is required for postnatal NSC activation and neurogenesis in the neurogenic niche. Here, we show that TLX modulates bone morphogenetic protein (BMP)-SMAD signaling to control the timing of postnatal astrogenesis. Genes involved in the BMP signaling pathway, such as *Bmp4*, *Hes1*, and *Id3*, are upregulated in postnatal brains lacking *Tlx*. Chromatin immunoprecipitation and electrophoretic mobility shift assays reveal that TLX can directly bind the enhancer region of *Bmp4*. In accordance with elevated BMP signaling, the downstream effectors SMAD1/5/8 are activated by phosphorylation in *Tlx* mutant mice. Consequently, *Tlx* mutant brains exhibit an early appearance and increased number of astrocytes with marker expression of glial fibrillary acidic protein (GFAP) and S100B. Taken together, these results suggest that TLX tightly controls postnatal astrogenesis through the modulation of BMP-SMAD signaling pathway activity.

## Introduction

Neurons and glia in the developing mammalian brain are derived from neural stem cells (NSCs) that reside in the ventricular zone (Temple, 2001; Kriegstein and Alvarez-Buylla, 2009). The “neuron first, glia second” sequential production of these cells is critical to central nervous system architecture and function (Miller and Gauthier, 2007). For instance, the delayed or precocious differentiation of astrocytes can contribute to dysfunctions of synaptic plasticity and neuropsychological disorders (Ullian et al., 2001). Accumulating evidence indicates that the Notch signaling (Morrison et al., 2000; Miller and Gauthier, 2007), Janus kinase-signal transducer and activator of transcription (JAK-STAT) signaling (Bonni et al., 1997; He et al., 2005), and bone morphogenetic protein (BMP)-SMAD signaling (Gomes et al., 2003; Guillemot, 2007) pathways control the appropriate timing of astrogenesis. How components of these pathways are molecularly regulated is not well-understood.

The orphan nuclear receptor TLX (also known as NR2E1) regulates NSC maintenance, self-renewal, and neurogenesis in both the embryonic and adult brain (Yu et al., 1994; Land and Monaghan, 2003; Roy et al., 2004; Shi et al., 2004; Li et al., 2008; Liu et al., 2008; Zhang et al., 2008). *Tlx* expression starts at embryonic day 8 and is restricted exclusively to the neuroepithelium of the developing mouse forebrain (Yu et al., 1994; Monaghan et al., 1995). Deletion of *Tlx* leads to a reduced thickness of the cerebral hemispheres, defects in retinal development, and violent behaviors (Monaghan et al., 1997; Yu et al., 2000; Zhang et al., 2006). In the subventricular zone (SVZ) of neonatal mice *Tlx* can be detected in both activated NSCs and their transit amplifying progenitors and plays an essential role for activation and differentiation of NSCs in this neurogenic niche (Obernier et al., 2011). TLX-dependent adult neurogenesis has a critical role in normal spatial learning and memory (Zhang et al., 2008). TLX-regulated proliferation of NSCs is also required for gliomagenesis in the adult neurogenic niches (Zou et al., 2012). TLX controls NSC quiescence and positioning in the neurogenic niche by modulating gene expression in multiple pathways including p53-p21 (Niu et al., 2011). In addition to its functions in neurogenesis, TLX directly regulates the expression of the astrocyte-specific marker GFAP in the adult dentate gyrus and NSCs (Shi et al., 2004). Removal of *Tlx* also leads to increased expression of GFAP in transit amplifying cells (Obernier et al., 2011). These data indicate a potential role for TLX in the regulation of astrogenesis. However, it is unclear how TLX controls the genetic program leading to complete astrocyte development. Through histological and genome-wide gene expression analyses, we show that TLX specifically regulates the activity of BMP-SMAD signaling but not JAK-STAT signaling during early postnatal astrogenesis.

## Materials and methods

### Animals

The generation of *Tlx^LacZ/LacZ^* (also known as *Tlx****^−/−^***) (Niu et al., 2011) or *Nes*-GFP (Yamaguchi et al., 2000) mice have been previously described. All mice were housed under a 12 h light/dark cycle with *ad libitum* access to food and water in a controlled animal facility. No gender-associated mutant phenotype was observed; thus, both males and females were included in the analyses. Experimental protocols were approved by the Institutional Animal Care and Use Committee at UT Southwestern.

### Immunohistochemistry and imaging

Postnatal brains at the indicated development stages were extracted and fixed in 4% paraformaldehyde after transcardial perfusion. Brains were further post-fixed overnight and then cryoprotected with 30% sucrose in PBS at 4°C. Coronal sections were cut at 40 μm thickness with a sliding microtome. In preparation for immunostaining, sections were washed with PBS and blocked with 3% BSA in PBS. After overnight incubation with primary antibodies diluted in the blocking solution with gentle agitation at 4°C, sections were washed and incubated with corresponding Alexa Fluor dye-conjugated secondary antibodies (1:500, Molecular Probes). Nuclei were counterstained with Hoechst 33342 (Hst). The following primary antibodies were used: GFP (chick, 1:500, AVES), GFAP (mouse, 1:1000, Sigma-Aldrich), S100B (rabbit, 1:500, Swant), NeuN (mouse, 1:500, Chemicon), pSMAD1/5/8 (rabbit, 1:200, Cell Signaling), and pSTAT3 (phospho-Y705, rabbit, 1:100, Cell Signaling). Fluorescent images were acquired on a Zeiss LSM510 META confocal system. For statistical analysis, the total number of positive cells were counted through randomly captured images at least from 6 sections (at least 2 sections per mouse).

### NSC culture and immunocytochemistry

*Tlx*-positive NSCs were prepared from 6-to-8-week-old *Tlx^LacZ/+^* mice as previously described through β-gal-based sorting using the FluoReporter lacZ Flow Cytometry Kit according to the user's manual (Invitrogen) (Shi et al., 2004; Zhang et al., 2008). The NSCs were cultured in growth medium containing DMEM/F12 medium supplemented with N2 (Invitrogen), heparin (5 μg/ml, Sigma), EGF (20 ng/ml, Peprotech), and bFGF (20 ng/ml, Peprotech) (Zhang et al., 2008). When indicated, BMP4 (20 ng/ml) was added to the culture medium for 2 days or the specified duration. BrdU (10 μ M) was added 5 h before fixation to label dividing cells. BMP4-treated cells were then fixed with 4% paraformaldehyde, washed with PBS, blocked for 30 min at room temperature (RT), and incubated overnight with primary antibodies in blocking solution at 4°C. BrdU-labeled cells were treated with 2 M HCl at 37°C for 30 min, washed with PBS, and incubated with primary antibodies.

### Gene expression by RNA sequencing (RNA-SEQ) and quantitative RT-PCR (qRT-PCR)

RNA-Seq analysis of purified GFP^+^ cells from the lateral ventricles of 3-week-old *Nes*-GFP mice has been previously described (Niu et al., 2011). Total RNA was isolated for qRT-PCR from independent samples in triplicate using the TRIzol protocol (Invitrogen) and cDNAs were generated using the SuperScript First-Strand cDNA Synthesis System (Invitrogen). qPCR was performed using iQ SYBR Green Supermix (Bio-Rad) and an ABI 7900HT instrument. The qPCR program was as follows: (step 1) 2 min at 50°C, (step 2) 10 min at 95°C, (step 3, 40 cycles) 15 s at 95°C transitioning to 60 s at 58°C. Primer quality was assessed using dissociation curves. The relative expression of each gene was analyzed using the ΔΔC*_T_* method (where C*_T_* is threshold cycle) using *Hprt* as a housekeeping gene.

### RNA *in situ* hybridization

*In situ* hybridization was performed as previously described (Qin et al., 2011). In brief, mouse *Bmp4* (803 bp) and *Id3* (790 bp) cDNA templates were generated by PCR and subcloned into the *pGEM-Teasy* vector. Digoxin-labeled sense or antisense riboprobes were generated by *in vitro* transcription with SP6 or T7 RNA polymerase (Roche). *In situ* hybridization was performed on coronal cryostat brain sections of 16 μm thickness.

### Electrophoretic mobility shift assay and chromatin immunoprecipitation (ChIP)

The synthetic oligonucleotides (5′-GCC CAT AGC CAT CAG TCA CAA CTA CCA-3′) containing one TLX-binding site (5′-CAG TCA-3′) were annealed and labeled with ^32^P-dCTP as a probe. Gel-shift assays were performed essentially as previously described (Zhang et al., 2006; Qin et al., 2013). Briefly, *in vitro* translated TLX protein was incubated with ^32^P-labeled probes (5 × 10^5^ c.p.m.) in binding buffer for 20 min at RT. Antibody-dependent supershift assays were performed by adding 1 μg rabbit anti-HA antibody (Santa Cruz) followed by incubation for 15 min at RT. An equal amount of normal rabbit serum was included as a control. Non-labeled double strand oligonucleotides were used for competition assays. ChIP assays were performed using NSCs transduced with retrovirus expressing either GFP or HA-tagged TLX. NSCs were fixed with 1% formaldehyde for 10 min at RT and the reaction quenched with 0.25 M glycine for 5 min at RT. ChIP was performed as previously described using a rabbit anti-HA antibody (Santa Cruz) (Qin et al., 2013). Purified DNAs were amplified with the following primer pairs: 5′-TTG TGA CTG ATG GCT ATG GG-3′ and 5′-AAG GGA TTG TGT CTC GCA TAC-3′ for the TLX-binding site and 5′-GGG TTT CAA AGG ATG GTC AAT G-3′ and 5′-AGG CTG GTC TCA AAC TTC TG-3′ for a distal control site.

### Statistical analysis

Data are expressed as means ± standard deviations. Statistical significance was determined using an unpaired Student's *t*-test with a *P* < 0.05 considered significant.

## Results

### TLX controls the timing of postnatal astrogenesis

**Figure 1:**
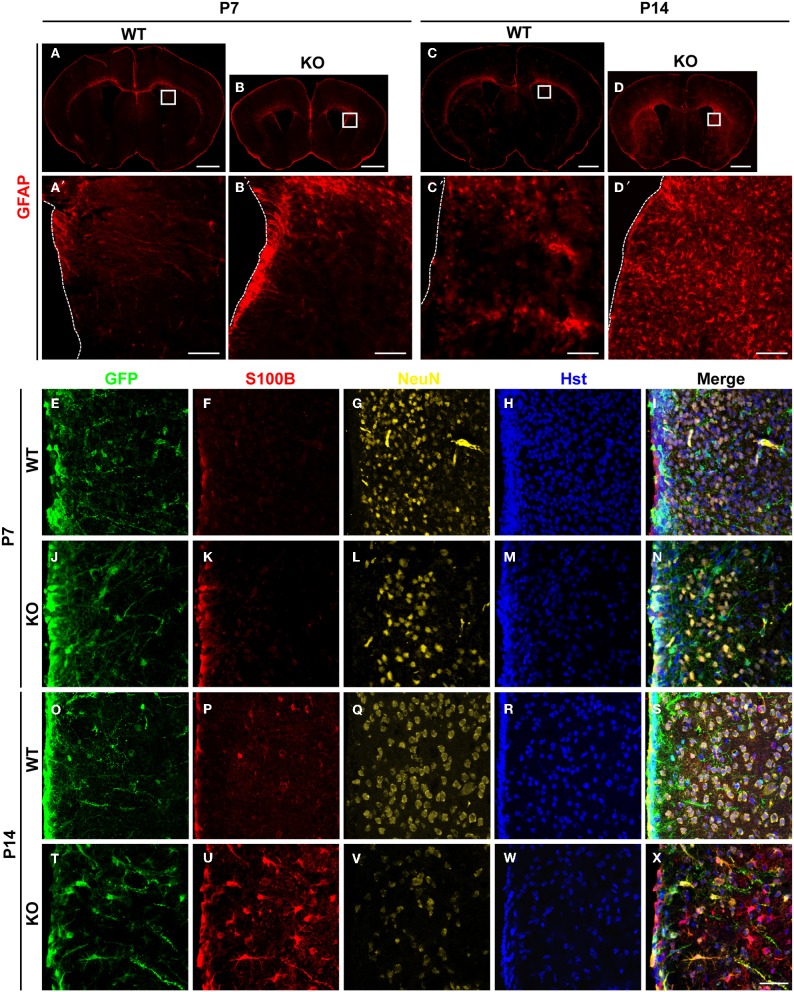
**Enhanced postnatal astrogenesis in *Tlx^−/−^ brains***. **(A–D)** GFAP expression in brains of *Tlx****^−/−^*** (knockout, KO) and littermate control (wild-type, WT) mice at P7 and P14 (*n* = 3). Higher magnification views of the boxed regions are shown on the lower panels **(A′–D′)**. The dotted lines show lateral ventricles (LV). **(E–X)** Expression of markers for astrocytes (S100B) and neurons (NeuN) during early postnatal stages. Deletion of *Tlx* leads to increased S100B^+^ astrocytes with a concomitant reduction of NeuN^+^ neurons. NSCs and their early progeny in the lateral ventricles are marked by *Nes*-GFP. Nuclei are counterstained with Hst (*n* = 3). Scales: 1 mm **(A–D)**, 100 μm **(A′–D′)**, and 50 μm **(E–X)**.

The number of astrocytes in the adult dentate gyrus of *Tlx****^−/−^*** brains is significantly increased suggesting potential roles for TLX in cell fate determination (Shi et al., 2004). To investigate this phenomenon, we conducted a detailed analysis of TLX function during the process of astrogenesis. Both radial glial cells and mature astrocytes express GFAP during early brain development (Doetsch, 2003). GFAP expression was slightly elevated around the lateral ventricular walls of *Tlx****^−/−^*** mice as compared to wild-type littermate controls at postnatal day 7 (P7) (Figures 1A,A′,B,B′). One week later (P14; Figures 1C,C′,D,D′), as many as two-fold more cells in the striatum of *Tlx****^−/−^*** brains strongly expressed GFAP compared to those in the same region of age-matched controls (625 ± 22 vs. 287 ± 7 cells/mm^2^; *p* = 0.0001, *n* = 3). These GFAP^+^ cells had a star-like morphology typical of astrocytes (Figure 1D′). The increased number of GFAP^+^ cells after *Tlx* deletion persisted into later postnatal stages such as P21 and P120 (data not shown).

The essential roles of TLX in neurogenesis and postnatal NSCs led us to examine the early fate specification of *Tlx****^−/−^*** stem cells using the *Nes*-GFP transgenic line, in which NSCs and their early progeny express GFP under the nestin (*Nes*) promoter (Yamaguchi et al., 2000). GFP^+^ cells were mainly located along the lateral ventricular walls with no significant difference observed between *Tlx****^−/−^*** mice and littermate controls at P7 (Figures 1E–N). The number and distribution of cells expressing S100B, a marker for differentiated astrocytes, was also similar between the groups at this early postnatal stage (Figures 1F,K). By P14, however, the population size of S100B^+^ cells in the striatum adjacent to the lateral ventricle of *Tlx****^−/−^*** mice increased more than four-fold compared to their littermate controls (Figures 1O–X; 578 ± 22 vs. 126 ± 11 cells/mm^2^; *p* = 0.0001, *n* = 3). Consistent with prior finding that *Tlx* regulates the timing of embryonic neurogenesis (Roy et al., 2004; Li et al., 2008), the density of NeuN^+^ neurons (Figures 1G,L,Q,V) in the striatum of *Tlx****^−/−^*** mice was reduced more than 50% compared to their wild-type littermate controls (1172 ± 23 vs. 2593 ± 20 cells/mm^2^ at P14; *p* < 0.0001, *n* = 3). Together, these data imply a critical regulatory role for TLX during both neurogenesis and astrogenesis in early postnatal brain development.

### TLX modulates the expression of genes involved in BMP signaling

**Figure 2:**
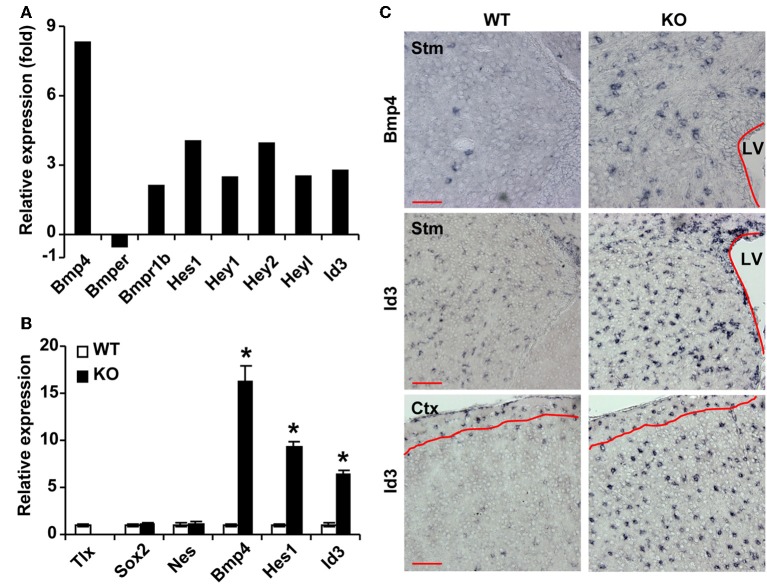
**TLX regulates genes involved in BMP signaling. (A)** RNA-Seq analysis of gene expression from sorted P21 *Nes*-GFP^+^ cells. **(B)** qRT-PCR analysis of selected genes using independent total RNA samples from sorted *Nes*-GFP^+^ cells. The expression of *Sox2* and *Nes* does not change. ^*^P < 0.001 by Student's *t*-test (*n* = 3). **(C)** RNA *in situ* hybridization confirms expression of selected genes at P14. The lateral ventricles (LV) are outlined. Red lines mark layer I where the expression of *Id3* is unchanged. Stm, striatum; Ctx, cortex. Scale: 100 μm.

To understand the molecular underpinnings of TLX-regulated astrogenesis, we performed whole genome expression analysis as previously described (Niu et al., 2011). GFP^+^ cells were isolated from the lateral ventricles of 3-week-old *Nes*-GFP transgenic mice crossed to either wild-type or *Tlx****^−/−^*** background mice. This later stage was selected to facilitate microdissections of the lateral ventricles for fluorescence-activated cell sorting based on GFP. Gene expression in these cells was determined by RNA-Seq (Niu et al., 2011). We focused on genes with known roles in astrogenesis and found that several genes in the BMP signaling pathway are differentially expressed (Figure 2A). The ligand *Bmp4* and the receptor *Bmpr1b* (also known as *Alk6*) are upregulated in *Tlx****^−/−^*** cells, whereas *Bmper*, an inhibitor of BMP4 signaling, is downregulated. Accordingly, the expression of several BMP downstream targets including *Hes1* and *Id3* (Nakashima et al., 2001; Dahlqvist et al., 2003; Mira et al., 2010) are also altered. These expression changes were further confirmed by qRT-PCR analysis of independently isolated total RNA from *Nes*-GFP^+^ cells (Figure 2B). By comparison, the NSC markers *Sox2* and *Nes* were not markedly altered, in line with the fact that the cells were *Nes*-GFP^+^ from the lateral ventricles. These qRT-PCR results on stem cell markers are consistent with what have been previously reported through RNA-Seq analysis (Niu et al., 2011), suggesting that cell sorting based on *Nes*-GFP isolated a similar population of cells from the *Tlx****^−/−^***and their wild-type controls. We next performed RNA *in situ* hybridization assays to examine the cellular level of BMP signaling in P14 *Tlx****^−/−^*** mice. Consistent with the results from RNA-Seq and qRT-PCR analyses, quantification showed a 3.5-fold increase of *Bmp4-*expressing cells in the lateral ventricles and striata of *Tlx****^−/−^*** mice as compared to littermate controls (Figure 2C; 256 ± 16 vs. 74 ± 12 cells/mm^2^, *p* = 0.0007, *n* = 3). Similarly, *Id3*-expressing cells were also more robustly detected in the same region or in the cortical area of *Tlx****^−/−^*** mice (503 ± 23 vs. 364 ± 20 cells/mm^2^ in the lateral ventricle-striatal region, *p* = 0.01, *n* = 3; 309 ± 7 vs. 38 ± 4 cells/mm^2^ in the cortical layers II-V, *p* < 0.0001, *n* = 3). Together, these data indicate that a key function of TLX is to suppress the BMP signaling pathway.

### TLX binds to the enhancer region of *Bmp4*

**Figure 3:**
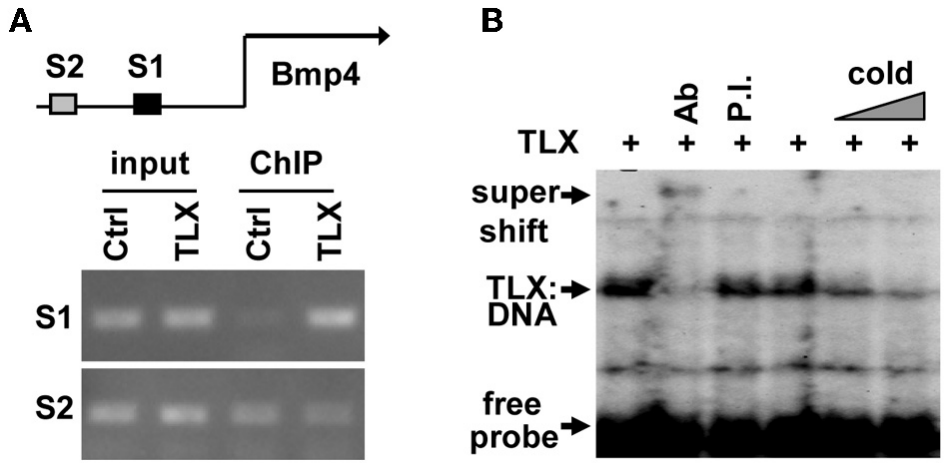
**TLX directly binds the enhancer region of *Bmp4*. (A)** ChIP analysis demonstrating the association of TLX with the *Bmp4* enhancer in NSCs. NSCs were transduced with retrovirus expressing either GFP (Ctrl) or HA-tagged TLX (TLX). The proximal S1 region (−4543 to −4383 bp from the transcription start site) contains a potential TLX-binding site, whereas the distal S2 site (−6468 to −6261 bp from the transcription start site) serves as a control. **(B)** Electrophoresis mobility shift assays showing direct binding of TLX to the S1 site shown in **(A)**. *In vitro* translated HA-tagged TLX was used. Ab, rabbit HA antibody; P.I., rabbit preimmune serum; Cold, unlabeled probe used as a competitor.

Close examination of the promoter and enhancer regions of the mouse *Bmp4* gene revealed several potential TLX binding sites. To demonstrate that TLX binds these sites, we performed chromatin immunoprecipitation (ChIP) assays. We detected specific binding of TLX to a site (5′-CAGTCA-3′) located at 4535 bp upstream of the transcription start site (−4535 bp) in the *Bmp4* enhancer using cultured NSCs infected with retrovirus expressing HA-tagged TLX (Figure 3A). We next performed electrophoretic mobility shift assays to confirm a direct association of TLX with the *Bmp4* gene. The mobility of a ^32^P-labeled DNA probe spanning the identified TLX-binding site was shifted by *in vitro* translated HA-TLX protein (Figure 3B). The addition of anti-HA antibody to the reaction yielded a supershifted band demonstrating that the DNA probe was specifically bound by TLX. Together, these results indicate that *Bmp4* is a direct downstream target of TLX, which acts to suppress BMP4 expression in NSCs. This TLX-mediated gene suppression is consistent with the demonstrated role of TLX as a transcriptional repressor (Yu et al., 2000; Shi et al., 2004; Zhang et al., 2006, 2008; Sun et al., 2007; Yokoyama et al., 2008).

### BMP4 promotes astrocyte differentiation *in vitro*

**Figure 4:**
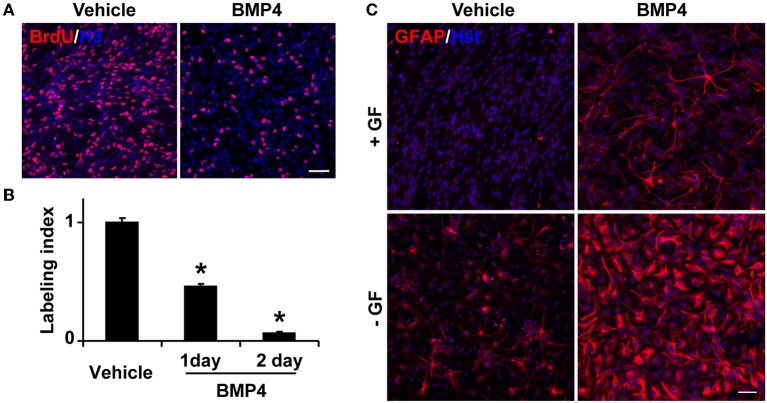
**BMP4 inhibits proliferation and promotes astrogenesis of cultured NSCs. (A)** Representative BrdU-labeled cells treated with vehicle or BMP4. Nuclei were stained with an antibody against histone H3 (H3). **(B)** The labeling index was calculated based on the percentage of BrdU^+^ cells in BMP4- vs. vehicle-treated NSCs in growth media at the indicated time points. ^*^P < 0.0002 by Student's *t*-test (*n* = 3). **(C)** BMP4 induces massive production of GFAP^+^ cells with astrocyte-like morphology. NSCs in culture medium with or without growth factors (GF) were treated with BMP4 or vehicle for 4 days. Growth factor-withdrawal and BMP4 synergize to induce differentiation. Scale: 250 μm.

To test whether BMP signaling regulates the behavior of *Tlx^+^* cells, we isolated *Tlx^+/LacZ^* NSCs from adult mouse brains by fluorescence-activated cell sorting based on *LacZ* expression (Zhang et al., 2008) and treated these cells with BMP4 (20 ng/ml) in the presence of growth factors. Consistent with previous observations (Yu et al., 2000; Bonaguidi et al., 2008; Mira et al., 2010), BMP4 treatment caused a significant reduction in the number of proliferating cells (Figure 4A). Approximately 90% of cells ceased to proliferate after 2 days of BMP4 treatment (Figure 4B). The identity of these BMP4-treated *Tlx^+^* cells was next examined by GFAP staining. In agreement with the known roles for BMP signaling in gliogenesis, we observed a robust induction of GFAP^+^ cells, which was undetected in vehicle-treated NSC controls under identical culture conditions (Figure 4C). The withdrawal of the growth factors bFGF and EGF led to spontaneous differentiation of NSCs into GFAP^+^ cells. However, the number of GFAP^+^ cells and the staining intensity were greatly enhanced by BMP4 treatment (Figure 4C). These data indicate that BMP4-dependent signaling plays a dominant role in astrocyte differentiation of cultured *Tlx^+^* NSCs.

### TLX modulates BMP-SMAD signaling *in vivo*

**Figure 5:**
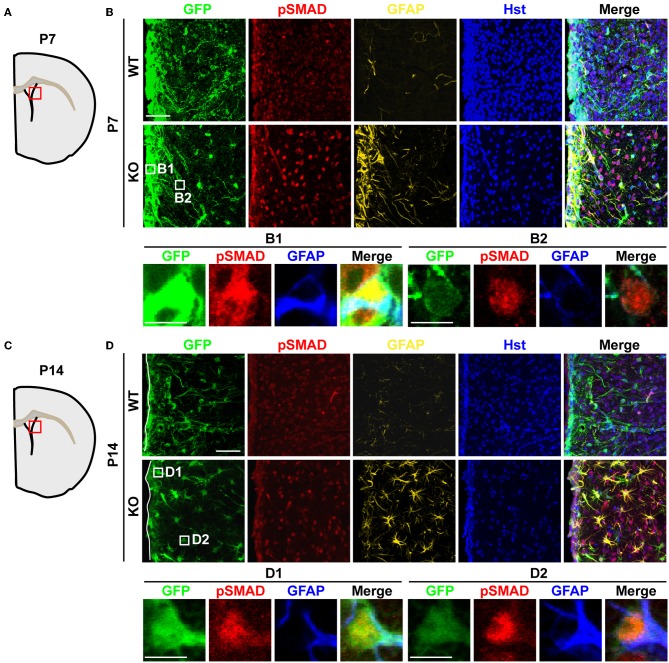
**Elevated BMP-SMAD signaling in *Tlx*-mutant brains. (A,C)** Schematic diagrams depicting the brain regions analyzed. **(B,D)** Increased immunoreactivity of both pSMAD and GFAP in regions of the striatum and lateral ventricle of *Tlx*-mutant brains at P7 and P14 (*n* = 3). NSCs and their early progeny are marked by *Nes*-GFP. *Tlx*-mutant mice also have much decreased cell densities (Hst^+^) reflecting a deficit in neurogenesis. Enlarged views of the boxed regions are also shown. Scales: 50 μm **(B,D)** and 10 μm **(B1,B2,D1,D2)**.

The binding of BMPs to their cognate receptors leads to the phosphorylation of intracellular SMAD1/5/8, which each associates with the common binding partner SMAD4. This SMAD complex then translocates into the nucleus to regulate gene expression and astrocyte development during perinatal stages (Attisano and Wrana, 2000). To examine whether increased BMP4 expression in *Tlx****^−/−^*** brains could lead to the functional activation of downstream signaling *in vivo*, we investigated the phosphorylation status of SMAD1/5/8 (pSMAD) and the expression of GFAP, a direct downstream target of BMP signaling (Nakashima et al., 1999, 2001). We observed a much stronger immunostaining signal for pSMAD in the striatum of P7 *Tlx****^−/−^*** mice as compared to controls (Figures 5A,B). This elevated signal is accompanied by the appearance of GFAP^+^ cells in the same region. At this early postnatal stage, the majority of GFAP^+^ cells were located adjacent to the lateral ventricle and labeled with *Nes*-GFP suggesting these cells might originate from differentiating NSCs (Figures 5B1,B2). Increased BMP-SMAD signaling in *Tlx****^−/−^*** mice persisted into later postnatal stages such as P14 (Figures 5C,D). GFAP^+^ cells with an astrocyte-like morphology showed enhanced nuclear pSMAD staining and were abundantly distributed in the striatal region of *Tlx****^−/−^*** mice. Cells close to the lateral ventricle also showed stronger *Nes*-GFP expression suggesting that these cells were still undergoing the transition from stem cells to astrocytes (Figures 5D1,D2). In contrast, wild-type controls had fewer GFAP^+^ cells with a much weaker pSMAD staining signal. These results suggest that BMP-SMAD signaling is tightly modulated by TLX to prevent the precocious differentiation of astrocytes during early postnatal development. It should be noted that numerous GFAP^−^ cells also showed elevated pSMAD staining in the striatal region adjacent to the lateral ventricle, most likely due to a non-cell autonomous effect of the increased BMP signaling on neighboring cells.

**Figure 6:**
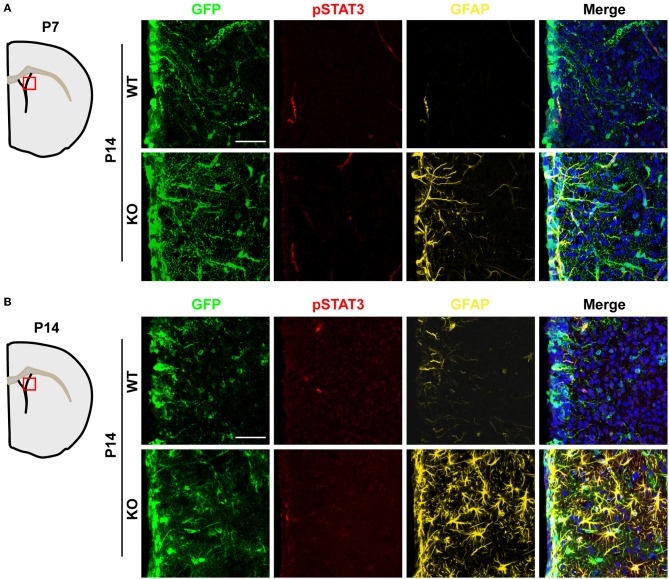
**Unaltered JAK-STAT3 signaling in *Tlx*-mutant brains**. JAK-STAT3 signaling was monitored by immunohistochemistry with an antibody for phosphorylated STAT3 at tyrosine 705 (pSTAT3). A similar pSTAT3-immunoreactivity was observed in both *Tlx*-mutant and control brains at both P7 **(A)** and P14 **(B)** (*n* = 3). Scales: 50 μm.

Activation of the JAK-STAT3 pathway in NSCs promotes astrogenesis (Bonni et al., 1997; Nakashima et al., 1999; He et al., 2005). Therefore, we evaluated whether TLX might also regulate this signaling pathway. We performed immunohistochemistry using an antibody specific for phosphorylation of the STAT3 residue tyrosine 705 (pSTAT3), a key effector of the JAK-STAT pathway during gliogenesis (Bonni et al., 1997; He et al., 2005). The specificity of an antibody recognizing pSTAT3 had been previously examined by western blot analyses and immunohistochemistry (Qin and Zhang, 2012; Qin et al., 2013). Interestingly, *Tlx****^−/−^*** and wild-type mice exhibited minimal staining for pSTAT3 in the lateral ventricle and the adjacent striatal region when examined at either P7 or P14 (Figures 6A,B), indicating that TLX is not a major regulator of the JAK-STAT3 signaling during postnatal astrogenesis.

## Discussion

**Figure 7:**
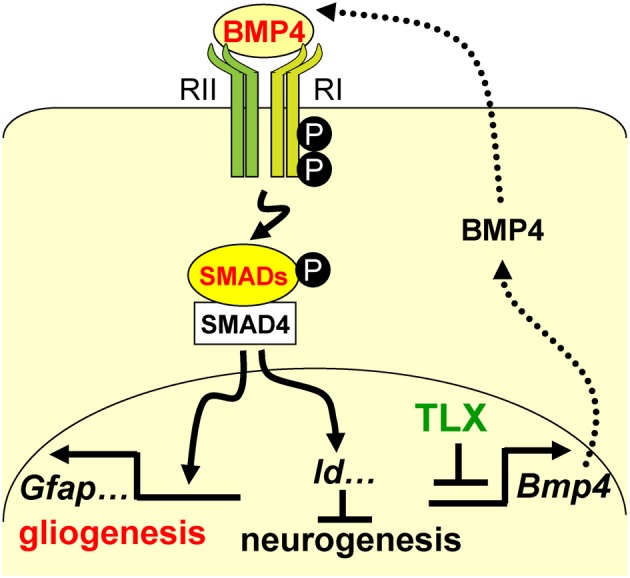
**A schematic diagram illustrating the interplay between TLX and the BMP-SMAD signaling pathway in the regulation of neurogenesis and astrogenesis**. TLX directly modulates the expression of the BMP ligands, which bind and activate the type I (RI) and type II (RII) receptors. These events result in phosphorylation of regulatory SMADs and their dimerization with the common cofactor SMAD4. The SMAD complex acts as a transcriptional activator to induce the expression of downstream targets, which promote astrogenesis and inhibit neurogenesis.

A precise balance between neurogenesis and astrogenesis is critical to the development of a functional central nervous system. Our studies show that the orphan nuclear receptor TLX controls the timing of postnatal astrogenesis by modulating the BMP-SMAD signaling pathway. This finding provides new insights into the role of TLX in NSCs during brain development and a molecular pathway that coordinates both the neurogenesis and astrogenesis processes (Figure 7).

BMPs are members of the transforming growth factor beta (TGFβ) superfamily of signaling ligands. Through signal transduction into the nucleus and transcriptional regulation, the BMPs play dynamic roles in the process of NSC neurogenesis and astrogliogenesis (Bond et al., 2012). BMP-mediated signaling in the adult SVZ is essential to direct the immediate progeny of NSCs toward a neuronal fate (Colak et al., 2008). However, cultured neural progenitors from the SVZ treated with BMPs are preferentially differentiated into the astrocyte lineage (Gross et al., 1996). The overexpression of *Bmp4* in transgenic mice leads to the enhanced generation and maturation of astrocytes in the brain, demonstrating that BMP4 is indeed a central player in astrocyte specification (Gomes et al., 2003). Our whole-genome and qRT-PCR analysis of gene expression showed that *Bmp4* is markedly upregulated in *Nes^+^* cells from *Tlx****^−/−^*** mice. Moreover, we have provided molecular evidence that TLX directly binds the enhancer region of *Bmp4*. These data collectively implicate TLX in the suppression of precocious *Bmp4* expression in NSCs. Elevated BMP4 levels in *Tlx****^−/−^*** mice might contribute greatly to the early and robust differentiation of astrocytes, an idea consistent with the demonstrated function of BMP signaling during neural development (Gross et al., 1996; Gomes et al., 2003; Bond et al., 2012).

Receptor-regulated phosphorylation of SMAD1/5/8 (pSMAD) is an integral step in the BMP signaling pathway (Wrana, 2000). We found that pSMAD levels increased in P7 *Tlx****^−/−^*** striatum. At this early postnatal stage, GFAP^+^ cells are restricted to a region close to the lateral ventricular wall, although robust pSMAD-staining is more broadly distributed in cells including neurons. Both NSCs and differentiated cells express the BMP-specific type I and II receptors suggesting that increased *Bmp4* expression in *Tlx****^−/−^*** cells may activate downstream SMADs through both paracrine and autocrine signaling pathways (Gross et al., 1996; Mira et al., 2010). The exposure of neural progenitors to BMP ligands induces inhibitors of differentiation (*Ids*), such as *Id1* and *Id3*, which inhibit neuronal differentiation and promote astroglial commitment (Nakashima et al., 2001). *Id3* expression in *Tlx****^−/−^*** brains increased significantly indicating that *Id3* might act as an effector to promote astrogenesis and suppress neurogenesis. In parallel with previous reports, neuronal density is vastly reduced in *Tlx****^−/−^*** brains (Roy et al., 2004; Li et al., 2008).

TLX has been shown to directly suppress *Gfap* gene expression by binding its enhancer/promoter region (Shi et al., 2004). Our work demonstrates that TLX also controls upstream BMP signaling, which hints that the genetic program for astrogenesis is coordinately regulated. The activation of BMP signaling not only controls *Gfap* expression but a whole set of genes required for astrocyte differentiation and maturation (Bond et al., 2012). The retina is another region of the central nervous system where *Tlx* is robustly expressed (Yu et al., 2000; Miyawaki et al., 2004; Zhang et al., 2006; Sehgal et al., 2009). During the protracted period of retinogenesis, TLX appears to be important for retinal astrocyte development through interactions with the Sonic hedgehog and BMP signaling pathways (Miyawaki et al., 2004; Sehgal et al., 2009). The canonical WNT/β-catenin pathway is another signaling cascade modulated by TLX and affects the proliferation and self-renewal of NSCs (Qu et al., 2010). Interestingly, the WNT and BMP signaling pathways often crosstalk to regulate similar biological processes (Itasaki and Hoppler, 2010). Collectively, these results demonstrate that TLX tightly controls NSC behavior, such as proliferation, neurogenesis, and gliogenesis, through the modulation of multiple signaling pathways at several developmental stages and in discrete brain regions.

## Author contributions

Song Qin and Chun-Li Zhang conceived and designed the experiments. Song Qin, Wenze Niu, and Nida Iqbal performed the experiments. Derek K. Smith critically commented the manuscript. Song Qin and Chun-Li Zhang analyzed data and prepared the manuscript.

### Conflict of interest statement

The authors declare that the research was conducted in the absence of any commercial or financial relationships that could be construed as a potential conflict of interest.
